# Effectiveness of multifaceted and tailored strategies to implement a fall-prevention guideline into acute care nursing practice: a before-and-after, mixed-method study using a participatory action research approach

**DOI:** 10.1186/s12912-015-0064-z

**Published:** 2015-03-31

**Authors:** Helga E Breimaier, Ruud JG Halfens, Christa Lohrmann

**Affiliations:** Institute of Nursing Science, Medical University of Graz, Billrothgasse 6, 8010 Graz, Austria; Department of Health Services Research, CAPHRI, Maastricht University, Duboisdomein 30, 6229 GT Maastricht, The Netherlands

**Keywords:** Participatory action research, Before-and-after design, Effectiveness, Implementation strategies, Guidelines, Nursing, Fall prevention

## Abstract

**Background:**

Research- and/or evidence-based knowledge are not routinely adopted in healthcare and nursing practice. It is also unclear which implementation strategies are effective in nursing practice and what expenditures of time and money are required for the successful implementation of clinical practice guidelines (CPGs). The aim in this study was to assess the effectiveness and required time investment of multifaceted and tailored strategies for implementing an evidence-based fall-prevention guideline (Falls CPG) into nursing practice in an acute care hospital setting.

**Methods:**

A before-and-after, mixed-method design was used within a participatory action research approach (PAR). The study was carried out in two departments of an Austrian university teaching hospital and included all graduate and assistant nurses. Data were collected through a questionnaire, group discussions and semi-structured interviews. Qualitative data were content-analysed using a template based on the *Consolidated Framework for Implementation Research (CFIR),* which also served as a theoretical framework for the study. Quantitative data were descriptively analysed using appropriate tests for independent groups.

**Results:**

By applying multifaceted and tailored implementation strategies, the graduate and assistant nurses’ knowledge on fall prevention, how to access the Falls CPG and the guideline itself increased significantly between baseline and final assessment (p ≤ .001). Qualitative data also revealed an increase in participant awareness of fall prevention. A baseline positive attitude towards guidelines improved significantly towards the end of the project (p = .001). Required fall prevention equipment like baby monitors or one-way glide sheets were available for use and any required environmental adaptations, e.g. a handrail in the corridor, were made. Hospital nursing personnel (approximately 150) invested a total of 1192 hours of working time over the course of the project.

**Conclusions:**

Multifaceted strategies tailored to the specific setting within a PAR approach and guided by the *CFIR* enabled the effective implementation of a CPG into acute care nursing practice. Nursing managers now have sound knowledge of the time resources required for CPG implementation.

**Electronic supplementary material:**

The online version of this article (doi:10.1186/s12912-015-0064-z) contains supplementary material, which is available to authorized users.

## Background

The quote “Knowledge is not enough, we must apply it. Being willing is not enough, we must do” by the famous German poet Johann Wolfgang von Goethe highlights the fact that knowledge and scientific evidence must be put into practice. However, the implementation of evidence-based care into healthcare systems is not typically the norm [1]. This also applies to nursing, despite the increasing expectation, and in some countries even a legal obligation, to work according to research-based standards/knowledge [2,3] in order to promote positive patient outcomes [4,5]. After nearly four decades of work implementing research- and evidence-based knowledge into healthcare and nursing, there is still a notorious mismatch between target and current conditions [6-8]. This mismatch threatens the safety and quality of patient care [8] as it may lead to unnecessary suffering [9].

Patient falls is one example which can lead to serious physical consequences such as fractures, which carry the risk of invalidity or even mortality. Falls may also have psychological consequences, like fear of further falls or a loss of self-confidence, in turn leading to a reduction in social activities [10,11]. Subsequently, falls may cause prolonged hospitalisation and increased treatment costs. Falls are a common problem in hospitals [12,13], particularly in patients aged 65 and older [14]. Around 30% of all persons aged 65 or older suffer a fall each year [15,16]. Heinze et al. found that between 3.2% and 37% of patients fell during a hospital stay, depending on the department [17]. An Austrian prevalence study revealed in 2011 that 2.1% of all patients suffered a fall during their hospitalisation. Furthermore, in nearly 40% of the hospitalised patients, no fall-prevention measures were taken, and only a minimum (less than 10%) of measures to prevent fall-related injuries were reported for patients who had experienced a fall [14].

As long as research-based recommendations are not adopted and acted upon, potential positive effects on patient health outcomes, such as a reduction in fall incidents, cannot be realised [18]. To facilitate the delivery of evidence- and research-based nursing care, scientific knowledge is frequently translated into inter-/national clinical practice guidelines (CPGs), such as the evidence-based guideline “Fall prevention for older and elderly persons in hospitals and chronic care facilities” [19] (subsequently named the Falls CPG). This guideline has compiled research-based recommendations on fall-preventive measures and their effectiveness and was made available to hospital nursing staff in 2009 via the hospital’s intranet. A paper version was delivered to each ward and all nurses were obliged to read the Falls CPG and to confirm this by signature. Despite this, the guideline was not applied in daily nursing practice.

We already know from the literature that the publication of CPGs does not guarantee their implementation or application [20]. Summarising good quality research evidence on fall prevention is, according to Wadell, a necessary first step in an institution, but is not sufficient in and of itself to effect change [21]. It should also be considered that the implementation of nursing guidelines in a hospital setting can be arduous [22] and presents a considerable challenge [23]. Numerous hindering factors with regard to the intervention itself, the context, characteristics of the individuals involved and the particular process level [24] may interfere with successful implementation.

As guideline implementation into a healthcare setting comprises various interconnecting steps and elements, it is generally considered to be a complex intervention [25,26]. Additionally, healthcare organisations are complex adaptive systems that encompass individuals who learn, interrelate, and self-organise to complete tasks. These individuals interact with their environment and consequently both, the respective system and the context, are reshaped through this interaction. Thus, by implementing an innovation like a Falls CPG, a healthcare organisation – or system – is reshaped and evolves over time. As these interactions are non-linear, their outputs are not entirely predictable [22,27]. Hence, a standardised approach is unsuitable and local circumstances must be taken into account when planning guideline implementation.

Even after having scrutinised scientific studies and systematic reviews, it remained unclear which strategies alone or in combination are the most effective in implementing guidelines into daily nursing practice and the circumstances under which it should be done [28,29]. Furthermore, most systematic reviews on the effectiveness of implementation strategies focus on medical doctors in primary care settings and healthcare personnel in general, often failing to specify the percentage of participating nursing staff. However, it must be noted that groups of health professional differ widely with regard to training, education, organisational structure and scope of practice and knowledge [30] and that the type of profession undoubtedly affects the intention to use CPGs in patient care [31], which is why implementation strategies that succeed in one profession may well fail in another. For this reason, the authors recommend using different strategies that target different professional groups [30,31].

Moreover, the use of multifaceted strategies [32,33] targeting existing barriers and other influencing factors – such as context, the innovation itself and characteristics of the professionals involved – are recommended in order to successfully meet the challenge of implementing guidelines into daily routine [24,34-37]. Tailored interventions that meet the contextual needs [38] can improve professional practice [39]. It can be concluded that multifaceted strategies tailored to the respective needs of a setting appear to be appropriate when implementing a CPG.

The consideration of cost and resource use is also important when implementing a guideline. Simpson and Doig recommend assessing the available resources when designing a change intervention [40], and Ploeg et al. emphasise recognising the ‘real’ costs associated with successful implementation of CPGs at the onset [41]. Nevertheless, the cost-effectiveness of guideline implementation strategies is rarely reported [32]. While keeping a budget-constrained health system in mind, it is crucial to consider the costs (and resources required) as well as the effects of an implementation [42].

### Scientific framework

The scientific framework of this study includes:

1. A participatory action research (PAR) approach [43-48].
2. The *Consolidated Framework for Implementation Research (CFIR)* [24,49].
  The CFIR formed the theoretical framework and underpinned the PAR approach, but each element influenced the other. The CFIR was used to identify influencing factors in an implementation project; the PAR approach, in turn, facilitated the identification of each procedural step in the implementation process, while taking into consideration the influencing factors.
3. The framework for implementation interventions/strategies provided by the *Cochrane Effective Practice and Organisation of Care* (*EPOC*) Review Group [50,51]. Mazza et al. defined an *implementation strategy* as “a purposeful procedure to achieve clinical practice compliance with a guideline recommendation” ([52], p. 1 of 10).

More information about the scientific framework is provided in Additional file 1.

### Aim

As it was unclear which implementation strategies are effective in nursing and what resources are required to implement a CPG, this study aimed to assess the effectiveness of multifaceted and tailored strategies in implementing an evidence-based fall-prevention guideline into nursing practice in an acute care hospital setting. Effective implementation was defined as an increase in the nursing personnel’s knowledge about fall-prevention measures; a positive change in their attitude towards evidence-based guidelines; and the fulfilment of successful implementation criteria as defined by the participants. The time invested by the nursing personnel in implementing the Falls CPG was of additional interest.

## Methods

### Design

A before and after, mixed-methods study was used within a PAR approach guided by the empirical-analytic approach according to Kemmis [48]. The *CFIR* [24] served as a theoretical framework for the study.

### Participants and setting

All graduate and assistant nurses (subsequently referred to as nursing personnel) of an Ophthalmic (OD) and Accident Surgery (ASD) Department of an Austrian university teaching hospital were included. Both departments were selected by the nurse director in agreement with all head nurses. Both clinics were deemed suitable for implementing the Falls CPG based on their patient clientele.

### Procedure

#### Informing nursing personnel

Nursing personnel were invited to attend a presentation on the aims and scope of the project and the study. These information sessions and the subsequent sessions on data collection were offered on different days of the week to accommodate participant work schedules. All participants received an information flyer including an informed consent form.

#### Data collection

**Figure 1:**
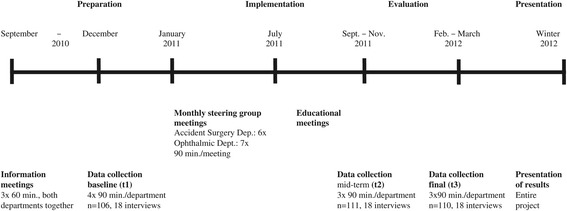
**Course of the project.**

Data were collected at three scheduled time points (t1 = baseline, t2 = mid-term, t3 = end of the project) through a questionnaire, guided group discussions and semi-structured interviews. Additionally, individual interviews were conducted at each data collection point. Details about the course of the project are outlined in Figure 1. Data collected at t1 and t3 were used to compare the main outcomes. As each data collection point focussed on different content, interview data from t2 are also included to illustrate the main outcomes.

#### Nursing personnel involvement

Nursing personnel involvement throughout the implementation process was guaranteed through (1) collaboration of both department representatives in steering groups held between January and July 2011; and (2) the opportunity to present ideas and/or critiques, either in person during a steering group meeting or indirectly through a representative. Furthermore, all parties were kept formally and informally up-to-date through steering group minutes sent to each unit and to the head nurse; through several meetings held with different combinations of involved persons; and via discussions between steering group members and their team. Further information detailing the steering group and its functions is provided in Additional file 2.

#### Implementation strategies applied

Six implementation strategies were tailored to the needs of each department, then applied and classified according to *EPOC* [50]: educational meetings, distribution of written materials, local opinion leaders, audit and feedback, adaptation of nursing record systems and changes in physical structure, facilities and equipment. A short description of each implementation strategy applied can be found in Additional file 2.

### Data collection tools

A structured questionnaire and semi-structured interview guidelines were used to obtain information about nurses’ knowledge of fall prevention and the Falls CPG; their attitudes towards evidence-based guidelines; and influencing factors based on the *CFIR*. The amount of total staff time invested in the implementation of the Falls CPG was consistently recorded in hours. The first investigator was responsible for recording the length of all research-related sessions, steering group meetings and interviews. The head nurse and steering group members were responsible for recording the amount of preparation and length of meetings held with ward nurses and for the educational sessions. The tools applied are described below.

#### Questionnaire

The same questionnaire relating to various aspects of the implementation project was employed for each collection of data. It captured demographic data, knowledge and attitudes. The influencing factors – i.e. competing values, self-efficacy, organisational learning, several characteristics of participating nursing personnel and, for the final assessment, the process evaluation – will be described in detail elsewhere. The final questionnaire was piloted on 22 graduate and assistant nurses working in two different departments at the same hospital. No major revisions were necessary. It took staff approximately 40 minutes to complete the questionnaire.

##### Demographics

The following demographic data were collected: age in years, gender, profession, year of diploma (<2001/≥2001), years of experience in current position, and full- or part-time employment status.

##### Knowledge

13 items were developed for this study that measured nursing personnel knowledge about the guideline in terms of risk of falls, fall prevention and recommended measures. The 7 single- and 6 multiple-choice items together contained 81 answer options. The questions were derived from the evidence-based guideline to be implemented and were drafted by HEB. They were refined within two Delphi rounds of four experts in the field, and pre-piloted with six students in a doctoral nursing programme. Internal consistency, measured with Cronbach’s alpha, was α (t1) = 0.69. It was also of interest to discover whether nursing personnel knew where to find the Falls CPG in the hospital intranet and by whom it had been developed.

##### Attitudes

The *Attitudes Towards Guidelines Scale (AGS)* consists of seven subscales: *general attitude, usefulness, reliability, lack of individual or team competence, lack of organisational competence, impracticality and availability* [53]. Each subscale consists of two Likert-scaled items from 1 = strongly disagree to 4 = strongly agree. The English version of the scale was translated into German by HEB, with the following two adaptations having been made: in one item, *medical practice* was replaced with *practice* and the specificity of *care providers* was increased by substituting it for *nursing personnel*. Since the original version was in Finnish, a Finnish nursing scientist with a good understanding of German translated the German version into Finnish. Both Finnish versions were compared by one of the developers who also gave permission to use the scale with its adaptions. The internal consistency (Cronbach’s alpha) of the seven subscales in the original study varied from 0.50 to 0.91 (sample 1) and from 0.42 to 0.79 (sample 2), respectively [53]. In the study by Alanen et al., Cronbach’s alpha varied from 0.68 to 0.74 [54]. In the current study (t1), it varied from 0.12 to 0.72. The total scale alpha, however, was 0.73.

#### Semi-structured interview guide

Interview guides for the semi-structured interviews and discussions were designed for each data collection point (t1 – t3). They were based on the CFIR framework and featured open-ended questions pertaining to each respective implementation stage. At the baseline, the aim was to assess influencing factors for the intervention; i.e.: intervention characteristics (e.g. familiarity with the Falls CPG content), inner and outer settings (e.g. local workflow) as well as characteristics of the individuals (e.g. participants’ self-efficacy). The *process* domain, with questions focussing, for example, on impact of the Falls CPG on nursing personnel’s daily work, satisfaction with the goals achieved or with the implementation strategies, was introduced at t2 and was the main focus at t3.

### Data analysis

Descriptive analyses (mean, standard deviation, percentages, frequency count) were performed with IBM SPSS Statistics 18. The mean values and standard deviations for the AGS composite score, its subscales and single items were calculated after all negatively keyed items had been reversed, meaning that higher scores express a more positive attitude. For comparison purposes, chi-square was used for categorical and dichotomous variables and t-tests for continuous variables. Analysis of dependent-group tests was not possible because the same participants did not always take part in each of the three data collection points. Furthermore, despite having been prompted, participants rarely marked their questionnaire with a traceable personal identifier. Significance level was set at 0.05. Qualitative data were content-analysed and managed in MAXQDA 10, a computer-assisted qualitative data analysis software. The CFIR, supplemented with four constructs (established fall prevention measures and implementation strategies; participant aims and wishes), provided a template for the analysis. The time invested was added to the length of information sessions, steering/group meetings and educational sessions and was multiplied by the respective number of participants.

### Ethical approval

Ethical approval was obtained from the university’s Research Ethics Committee (EK-No. 21–334 ex 09/10) prior to initiating the study. All participants gave their written informed consent.

## Results

### Response rates

The response rates were 82.8% (n = 106) at t1 and 94.8% (n = 110) at t3. Questionnaires were excluded if the participant did not belong to a nursing profession (t1 & t3 each 1x), or if they were returned either empty (t1: 20 x; t3: 3x) or only partly filled in (t1: 1x; t3: 2x); individual missing answers were accepted.

### Demographics

Approximately two thirds of the participating nursing personnel worked in the OD (t1: 65.1%, n = 106; t3: 68.2%, n = 110). Participants were on average 38 years old (t1: 38.97, SD = 10.57, n = 102; t3: 38.27, SD = 10.42, n = 99), with the great majority being female (t1: 93.3%, n = 105; t3: 94.2%, n = 104). Nearly two thirds of the participants were employed as graduate nurses (t1: 66.3%, n = 104; t3: 74.3%, n = 105). More than 50% of the graduate nurses finished their educational training prior to 2001 (t1: 66.7%, n = 69; t3: 54.5%, n = 77), which was before the introduction of nursing science into the Austrian nursing curriculum. A large proportion of all participants had more than 10 years of experience in their current position (t1: 55.4%, n = 101; t3: 44.2%, n = 104) and approximately one third were employed part time (t1: 32.7%, n = 104; t3: 40.8%, n = 103).

### Main outcomes at the level of nursing personnel

#### Knowledge

**Figure 2:**
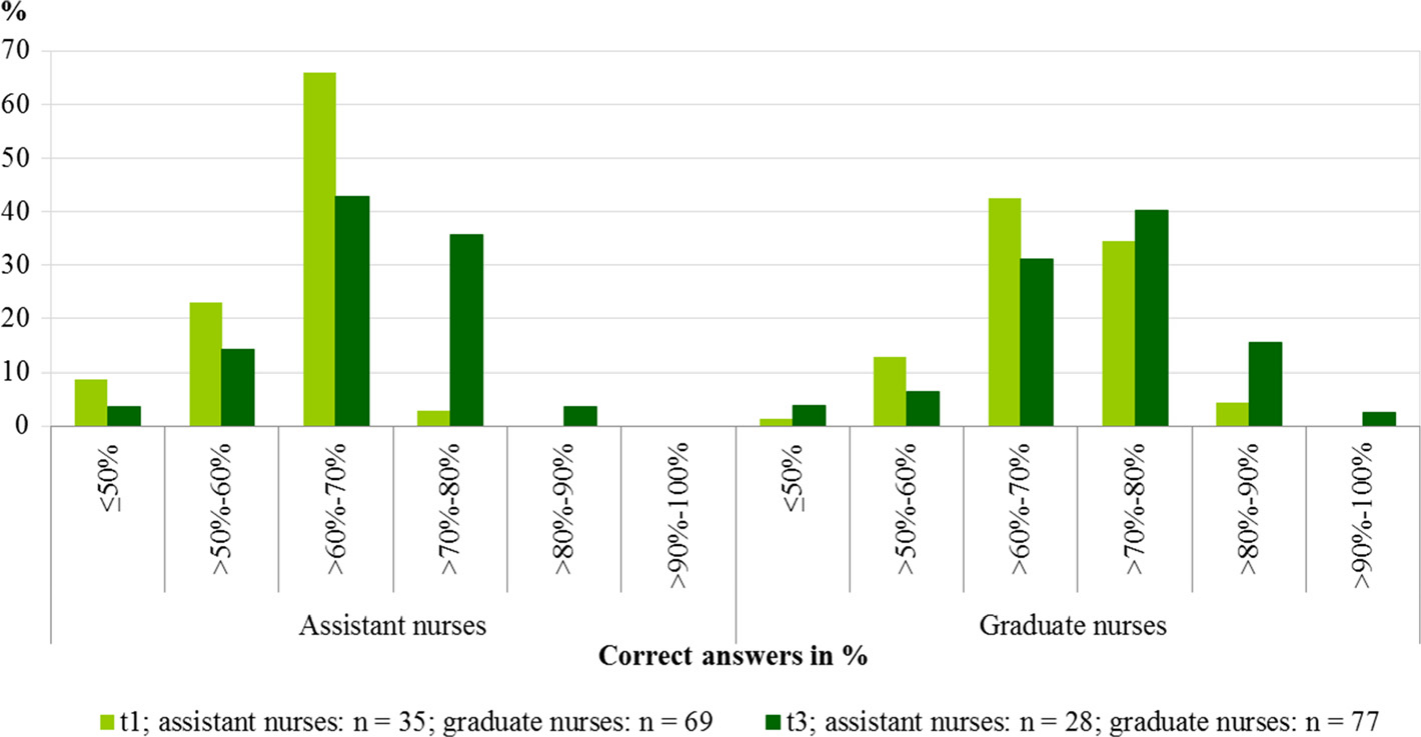
**Distribution of correct answers (in %) in assistant and graduate nurses at t1 and t3.**

Compared to t1, significantly more participants knew how to access the Falls CPG by the final data collection (t3). The proportion increased from 52.4% (n = 105) to 81.8% (n = 110, p < .001). Additionally, more participants knew by whom the Falls CPG had been developed: 61.5% (n = 104) compared to 37.4% (n = 99, p < .001). Nursing personnel knowledge about fall prevention and the Falls CPG improved significantly from 65.6% (SD = 8.221, n = 106) to 69.7% (SD = 9.150, n = 110, p = .001). This significant difference is particularly attributable to a knowledge gain in the group of assistant nurses. They showed an improvement from 61.3% (SD = 6.835, n = 35) to 68.0% (SD = 8.223, n = 28, p = .001) whereas in the group of graduate nurses the improvement lacked statistical significance: from 68.0% (SD = 7.821, n = 69) to 70.5% (SD = 9.189, n = 77, p = .072). Although at baseline both groups differed substantially in this regard (p ≤ .001), the resulting difference was equalised at t3: p = .201. Figure 2 shows the distribution of correct answers (in %) in assistant and graduate nurses at t1 and t3, respectively.

Qualitative data (t2, t3) revealed participants’ knowledge gain. The greatest and most beneficial change, as a result of this project, was perceived by participants as being their increased awareness of fall prevention. This was the most important issue of all discussions, meetings and interviews at t2 and t3. One participant stated that although there was nothing really new regarding the measures taken to prevent falls, the project had not been in vain:

*Preventive actions are now taken with increased awareness. Instructions and explanations for patients are given more conscientiously in order to achieve compliance. Nursing students are instructed more consciously about fall prevention* (t2_6_360).

The understanding of the significance of falls and fall prevention increased, and the understanding of the significance of restraints and their legal implications became clear (t2 & t3). This changed awareness also affected participant behaviour in that they reported, for example, having gained new insight into routine interventions which had up to that point been performed automatically. Present conditions began to be scrutinised and were no longer blindly accepted (t2). In the event of a patient’s fall, graduate nurses learned how to fill in a detailed report form (t3). They also realised (t2 & t3) that fall assessment and documentation, as well as patient information regarding fall prevention, were to be performed in a standardised manner, with a clear focus on consistency and coherence. Furthermore they realised that the Falls CPG included useful recommendations for each field of work and that these recommendations were helpful in decision making.

#### Attitudes

The nursing personnel generally displayed a positive attitude towards guidelines from t1: mean = 3.014 (SD = .0353, n = 81), which actually improved significantly towards the end of the implementation project at t3: mean = 3.188 (SD = .0344, n = 101, p = .001). The values for the seven subscales and all individual items of the AGS are presented in Table 1. The most positive attitudes were related to the *general attitude toward guidelines* and the *usefulness of guidelines* throughout the project. At t1 and t3, guidelines were seen as improving the quality of healthcare, useful as an educational tool and a convenient source of advice. Furthermore, the nursing personnel denied not having seen any guidelines in their healthcare unit. A significant improvement in attitude between t1 and t3 occurred regarding *impracticality* and *availability of the guidelines* (p ≤ .001). The attitudes regarding two single items remained negative throughout the implementation project: the nursing personnel believed that most of their team members harboured disapproving attitudes about guidelines and that they would oversimplify nursing practice (Table 1). However, with respect to one item, the nursing personnel’s attitude changed from a negative to a positive one: by t3, they had stopped believing that guidelines challenged their autonomy.

**Table 1:** **Nursing personnel’s attitudes toward guidelines (range 1–4, with higher scores signifying more positive attitudes)**

**Attitudes towards guidelines**	t1 n = 99-106*		t3 n = 107-110*	
	**Mean**	**SD** ^**§§**^	**Mean**	**SD** ^**§§**^
*General attitude toward guidelines*	*3.43*	*0.537*	*3.40*	*0.527*
1. Guidelines are useful as educational tools.	3.43	0.589	3.41	0.610
2. Guidelines are a convenient source of advice.	3.44	0.619	3.40	0.578
*Usefulness of guidelines*	*3.30*	*0.502*	*3.31*	*0.546*
3. Guidelines can facilitate communication with patients and families.	3.12	0.658	3.20	0.677
4. Guidelines can improve the quality of healthcare.	3.46	0.574	3.40	0.610
*Reliability of guidelines*	*3.10*	*0.696*	*3.23*	*0.728*
5. Guidelines are based on scientific evidence.	3.11	0.832	3.33	0.762
6. Guidelines are made by experts.	3.07	0.915	3.12	0.900
*Lack of individual or team competence*	*2.88*	*0.596*	*2.99*	*0.612*
7. My occupational competence is sufficient for adopting the latest guidelines.	3.30	0.698	3.35	0.599
8. Most of our team members have disapproving attitudes about guidelines.^§^	2.43	1.039	2.63	0.956
*Lack of organisational competence*	*3.01*	*0.601*	*3.15*	*0.614*
9. Guidelines are valued in our organisation.	3.06	0.651	3.18	0.722
10. Implementing guidelines is too expensive for us.^§^	2.96	0.803	3.12	0.832
Impracticality of guidelines**	*2.32*	*0.702*	*2.70*	*0.677*
11. Guidelines challenge the autonomy of nursing personnel.^§^	2.75	0.805	3.13	0.721
12. Guidelines oversimplify nursing practice.^§^	1.89	0.795	2.26	0.886
Availability of guidelines***	*3.15*	*0.643*	*3.42*	*0.540*
13. Guidelines are difficult to find if needed.^§^	2.69	0.841	3.00	0.828
14. I have not seen any guidelines in our healthcare unit.^§^	3.62	0.791	3.83	0.448

*Variation in number of respondents because of some missing answers; **p < .001; ***p = .001.

^§^Reversed item; ^§§^Standard deviation.

A further consequence of this project was extracted from the qualitative data: a strengthening of participants’ self-confidence. They felt a new ability to reasonably debate with third parties regarding fall prevention (t2). The debate about the Falls CPG changed their satisfaction with established practice and they reported realising what might be enhanced and improved (t2):

*I believe that everybody had heard something about the guideline at some point, and it was actually compulsory to read it. Well, at least the registered nurses* [were obliged to read it], *as far as I know. And somehow we believed that we were already able to do all this* [and] *we were already* [taking measures against fall prevention] *anyway. But then, only when we went into detail, it became apparent what we could improve upon and what we could pay more attention to. It was only at this point that it actually became important* (t2_9_76).

### Main outcomes at organisational and process levels

#### Availability of necessary means/equipment for fall prevention

The second most important benefit from the participants’ point of view was the availability of more fall-prevention devices, acquired to assist nursing personnel in daily practice, and some modifications of surroundings. A baby monitor is now in use to facilitate the monitoring of high-risk patients such as those suffering from dementia, especially during the night shift. Several devices for the safe and easy transfer of patients were obtained; for example a transfer board, one-way glide sheets and a transfer turntable. Additional walking aids were also acquired, such as wheelchairs and Zimmer frames, toilet seat raisers, commode chairs, gel cushions to prevent patients from slipping out of a wheelchair, and crutch holders for the beds. A manoeuvrable examination chair was purchased for the OD’s walk-in clinical department to avoid difficult transfer of wheelchair dependent patients. A handrail was installed in the ASD corridor. A nurse call was also installed on the balcony so that patients could ring for help to safely cross a doorframe ramp, which had been identified as risk factor. Moreover, written patient information materials for a planned hospital admission were updated.

#### Supplementary information folders

During the working-group meetings, supplementary information folders were compiled, which are now readily available to nursing personnel in each field. The folders contain the Falls CPG accompanied by concise information on fall-related risk assessment, a list of context-relevant risk factors and a list outlining groups of drugs with their potential to increase the risk of a fall, e.g. by causing dizziness. The folder also includes practical guidance and prompts on instructing patients as well as examples/illustrations of writing fall reports.

#### Participant feedback - process level

At the end of the implementation project, participants reported having valued the course taken; they found the change straightforward and comprehensible and the implementation easily applicable. It was emphasised that further implementation projects should be carried out in the same manner.

### Supplementary outcome at patient level

In this study, patient falls during hospitalisation would have been an appropriate outcome measure on a patient level. Unfortunately, fall incidences were not being consistently recorded and no valid baseline data were available. This fact hindered a before-and-after comparison. With the implementation of the fall-prevention guideline, however, the procedure for recording patient falls in the case of an incidence has been established. This will now be available for further evaluation not only within the two departments but also for the hospital.

### Resource use

In total, 1192 hours of working time were invested in the project by the hospital’s nursing personnel (details are presented in Table 2). Taking into account the basic wage of a graduate nurse with 10 years of work experience, this would amount to a project expense of about 14,600 €/11.600 £.

**Table 2:** **Resource use in terms of invested time of involved hospital nursing personnel**

**Type of resource use**	**Hours invested**
Participation in nursing personnel’s information (1 h/session)	110 h
Participation in group meetings – data collection (1 ½ - 2 h/session)	598 h
Participation in interviews (app. 0.75 h/session)	41 h
Steering group meetings (1 ½ - 2 h/session)	147 h
Preparation for steering group meetings, meetings with ward nurses and head nurse (0.5 – 4 h/session)	109 h
Educational sessions (app. 1.5 h/session)	187 h
**Σ**	**1192 h**

## Discussion

This study aimed to assess the effectiveness of multifaceted and tailored strategies in implementing an evidence-based Falls GPG into an acute care hospital nursing practice as well as to assess the time resources used. Using a participatory action research approach, six implementation strategies were determined by participants and tailored to the necessities of each department and its units. The strategies were tailored based on the assessment of possible hindering and facilitating factors guided by the *CFIR* and its supplementation. Study findings at the nursing personnel and organisational/process levels supported the successful implementation of the Falls CPG in the two participating departments, whereby the time invested turned out to be relatively low.

### Nursing personnel level

The multifaceted and tailored strategies improved nursing personnel’s knowledge of how to access the Falls CPG and how to prevent falls. With regard to the latter point, however, although the gain in knowledge was statistically significant, the effect of 4.1% can be regarded as low. Three factors may have contributed to this:

1. At the onset of the project nursing personnel’s knowledge was already satisfactory, and taking fall-prevention measures was routine practice. Dickinson et al. pointed out the importance of recognising *that it is often within the basics of care such as* [the prevention of falls] *that the rituals of nursing survive and changing practice in these areas requires the letting go of experiential knowledge built and handed on over many years* ([55], p. 40). Furthermore, it may be harder to substantially increase knowledge from a higher level starting point than from a lower one. This was supported by a closer examination of results. Applying the Dreyfus model from novice to expert adapted by Lester [56], the results revealed a remarkable shift between advanced beginner and fully competent assistant nurses; i.e. between having a working knowledge of key aspects of fall prevention to good working and background knowledge of fall prevention. The shift in graduate nurses, which mainly started with advanced beginners and competency and remained steady or moved to competency and proficiency, was less obvious. Nearly one third of participating graduate nurses remained advanced beginners and only a few achieved competency and proficiency. The latter indicates a depth of understanding of the discipline and area of practice [56], in this case related to fall prevention.
2. The fluctuation of nursing staff in the OD was exceptionally high due to the retirement of ward managers and staff nurses who were then replaced by younger and therefore less experienced personnel. This may explain the relatively high percentage of graduate nurses who remained advanced beginners.
3. Additionally, the following three hindering factors may have contributed to an overall low knowledge gain:
  The first factor may have been the reluctance of OD operation theatre participants based on reasons found within four domains of the *CFIR*: *Intervention characteristics* (no special focus on working with patients in an operation theatre)*, inner setting* (no perceived tension for change)*, characteristics of individuals* (no recent experience with falls) and *process* (the representative could not always participate in the steering committee meetings). Unless there is, according to Dickinson et al., *sufficient discomfort or risk associated with current nursing practice, it is unlikely that nurses will immediately see the need for change and respond to the introduction of a* CPG ([55], p. 40). The decision to include this unit into the implementation project was made by the hospital’s nursing management immediately before the start of the project. During the process, the head nurse acknowledged the fact that the Falls CPG was not equally relevant for all working areas within the OD.
  Secondly, the mode and content of the educational programme carried out in the OD was influenced by the head nurse and ward managers: It was carried out as a lecture for all nursing staff in one session and not, as previously intended, separately for each working unit. A further topic was included which, according to nursing personnel feedback, did not strongly grasp their attention, namely the hospital’s underlying nursing theory related to fall prevention.
  Thirdly, ASD’s *relative priority* (*implementation climate*) was also regarded as having been a limiting factor: Two other projects were being conducted at nearly the same time, or close to the start of this project, which limited attention and resources.

Apart from these potentially hindering factors, participants nevertheless appreciated the contribution of the Falls CPG implementation to their increased awareness of fall prevention and interventions formerly performed automatically and without reflection. It can be said that the project helped bring to light a certain part of the invisible mass of an iceberg, i.e. nursing personnel’s tacit knowledge that can be regarded as the ‘treasure in our heads’ [57]. This might also be of particular importance for nursing in German-speaking countries: verbalising/emphasising taken-for-granted areas of nurses’ work entails giving it a voice and making it visible, as only this enables the valuing of the practice of nursing both monetarily and with respect to the time invested . When the implementation of the Falls CPG began, participants pointed out that they already did so many things to prevent falls. However, the Austrian version of the *International Prevalence Measurement of Care Problems,* conducted in Austrian hospitals and nursing homes in 2011, revealed that almost no fall prevention measures were reported as being taken [14]. This still supports Abt-Zegelin’s statement that, for example, informing patients about risk factors in the hospital area is done ‘en passant’ leading to a lack of clarity about how to represent common nursing interventions in nursing documentation [58] and consequently in a survey where participants are asked to write down used measures.

Attitudes and beliefs are considered an important factor influencing implementation processes [54,55]. Similar to the study of Alanen et al. [54], nursing personnel participating in the present study showed positive attitudes towards guidelines and further improved them. These findings support Alanen et al.’s assumption that implementation interventions improve attitudes toward guidelines [54]. The nursing personnel’s retrospective feedback that the Falls CPG was now easily understandable also strengthens Alanen et al.’s assumption of positive attitudes improving familiarity with guidelines [54]. Although the nursing personnel remembered that they had once had to read the Falls CPG, and acknowledged having seen the guideline in their working unit, they seemed unable to remember where to find it on the hospital’s intranet. The multifaceted and tailored strategies helped the nursing personnel to improve their attitude regarding its availability. This is in line with the nursing personnel’s gain in knowledge as described above. Alanen et al. also noticed that nurses in implementer health centres saw guidelines as more available than nurses in disseminator centres [54].

Although the *impracticality of guidelines* remained negative at t3, there was a significant improvement towards the positive, especially with regard to one item: In light of this project, nursing personnel revised their opinion that guidelines challenged their autonomy. This change may be attributable to their involvement in the project and the educational programme, where they had been informed that recommendations from a CPG did not have to be followed blindly, but rather on the basis of their profound clinical judgement of a patient’s situation. The participants’ negative assumption that most of their team members harboured disapproving attitudes about guidelines may be explained by a discrepancy between what was articulated among the nursing personnel, for assumed reasons of social acceptance, and what each participant actually believed, as the overall attitude among participants was mainly positive. The majority of nursing personnel still believe that guidelines oversimplify nursing practice, although a certain degree of improvement was visible. This may be explained by the fact that patients and patient contacts are seen as highly individual. Guidelines are therefore regarded as never being able to fully illustrate the complexity of each individual patient situation. In practice, however, nurses have to make individual patient decisions, taking into consideration his/her needs, resources, and of course, external evidence.

Implementing the Falls CPG strengthened nursing personnel’s self-confidence. According to White, achieving self-confidence allows a more autonomous practice to be built which ultimately benefits the recipients of nursing care; and having self-confidence allows nurses to realise professional collaboration [59]. Participating nursing personnel confirmed that they now felt able to reasonably discuss fall prevention with third parties. The promotion of knowledge is one of the identified antecedents to the acquisition of self-confidence [59].

### Organisational or process level

Guideline recommendations can only be adhered to if the necessary means and resources are available. Within this implementation project, several diverse resources and equipment were purchased and installed. Additionally, a supplementary information folder was compiled for each working unit with the respective relevant information. Thus, successful implementation criteria from nursing personnel’s point of view were satisfyingly met even though no extra budget was provided for the implementation of the Falls CPG. In this respect it can therefore be argued that the approach to implementing the Falls CPG was effective.

All in all, this study strengthens results from existing literature [39,60,61], finding that multifaceted and tailored implementation strategies are an effective means to implement a CPG, not only into healthcare practice but also into acute care nursing. It is assumed that multifaceted strategies based on a diagnostic analysis are more effective than single strategies because, according to Hulscher et al., multiple barriers can be removed [61]. As requested by the authors, the choice of selected strategies in the implementation project depended on the results of a comprehensive diagnostic analysis. This should be carried out at the beginning of and throughout the implementation project through determining barriers and facilitators. PAR proved to be a helpful approach in carrying out this project and bringing it to a successful conclusion. Participants were satisfied with the approach and the obtained results.

### Resource use

The second aim of this study was to determine the resources required to implement the Falls CPG because hardly any study informs readers about what expenditures of time and money have to be calculated for implementing a CPG. The results demonstrate the real expenditure of time necessary to implement the Falls CPG, allowing one to calculate staff-related costs, among others, as requested by Ploeg et al. [41].

### Limitations

The following limitations have to be acknowledged: It was not possible to analyse the before and after changes with a paired t-test as intended. Participants were asked, as recommended by the consulted statistician, to mark their questionnaire with a traceable personal identifier. An example was given to allow its tracing during data collection while still maintaining anonymity: the initials of their mother’s birth name and the two last ciphers of her year of birth. However, about one third of participants recorded 99 as an identifier and a substantial number recorded a combination of letters and ciphers which they could not recall at the following data collection point. Only a minority (<20%) recalled their personal identifier correctly. It was therefore decided that unpaired t-tests should be used to compare before-and-after results. One of the greatest challenges in this PAR project was its duration. Due to unforeseen circumstances it took nearly 18 months to complete the study. Firstly, the beginning was delayed because another project within the OD had not been finished as planned. This caused a further delay of the mid-term assessment which could not be carried out immediately after finishing the working group meetings with less staff being available due to summer holidays. Thus, the mid-term assessment was only scheduled about three months before t3, which might have influenced the results. At the same time, this circumstance helped to keep the topic of fall prevention alive in nursing personnel’s daily work. Shortly before the start of the implementation project, nursing management decided to also include the OD’s operation theatre and its outpatient clinic. As the questionnaire did not specify the individual participant’s working area, it was not possible to analyse the potential effect of this aspect, especially with respect to OD’s operation theatre. Each completion of the questionnaire took about 40 minutes, which might have had a negative influence. The required time, however, was completed during work hours and approved as such. A clear strength of this study lies in the high response rate and the participatory approach.

## Conclusions

Overall, this investigation showed that multifaceted strategies tailored to a specific setting using a PAR approach and guided by the *CFIR* were an effective means to implementing a CPG into nursing practice in an acute hospital setting. Recommendations for further implementation projects are available and nursing managers now have sound knowledge about the time resources required to implement a CPG into acute care nursing practice.

## Additional files

Additional file 1:
**Scientific framework.** Description of the scientific framework used. Additional file 2:
**Nursing personnel involvement and applied implementation strategies.** Description of nursing personnel involvement during the implementation process and of the implementation strategies used.

## Abbreviations

AGS: Attitudes towards guidelines scale
ASD: Accident Surgery Department
CFIR: Consolidated Framework for implementation research
CPG: Clinical practice guideline
EPOC: Cochrane Effective Practice and Organisation of Care
i.e: that is to say
OD: Ophthalmic Department
PAR: Participatory action research
t1: Baseline data collection
t2: Mid-term data collection
t3: Final data collection
