# Supplementary material for: Effectiveness of multifaceted and tailored strategies to implement a fall-prevention guideline into acute care nursing practice: a before-and-after, mixed-method study using a participatory action research approach
Source: BMC Nurs. 2015 Mar 31;14:18. doi: 10.1186/s12912-015-0064-z (PMC4394413; doi:10.1186/s12912-015-0064-z)
Supplement: Additional file 1: — Scientific framework. Description of the scientific framework used. [file 12912_2015_64_MOESM1_ESM.doc]

**Additional file 1: Scientific framework**

Breimaier HE, Halfens RJG, Lohrmann C: **Effectiveness of multifaceted and tailored strategies to implement a fall-prevention guideline into acute care nursing practice: a before-and-after, mixed-method study using a participatory action research approach**

To achieve and ensure sustainable implementation, a ‘whole-systems’ approach like participatory action research approach (PAR) [1, 2] is recommended. Such a comprehensive approach takes into account all facets of a healthcare setting [2]. PAR involves a period of inquiry which describes, interprets and explains social situations/practice. A change intervention is then simultaneously adopted that aims to improve a practice situation and involve all persons concerned in the research process as collaborating partners of the researcher ([3], p. 11; [4], p. 183; [5]). Action research itself is a cyclical process of continuous assessment, planning, action and evaluation. This provides the opportunity for the procedures to be adapted to the existing needs of a given situation [6]. Such a flexible approach also masters the challenge of changing practice as it allows for assimilation of the unforeseeable through continuous observation and reflection [4]. According to Kemmis, its empirical-analytic approach is “oriented essentially towards functional improvements in terms of its success in changing particular outcomes of practices” and “an interest in getting things done effectively” ([7], p. 95); or, as Smith expressed, it “is concerned with testing [the] effectiveness of an intervention” ([5], p. 85). A PAR approach focuses on essential features that until now have rarely been considered in the process of implementing guidelines or research results into healthcare settings.

The *Consolidated Framework for Implementation Research (CFIR)*, developed by Damschroder et al. [8], focuses on systems as a whole [9] and thus seemed to fit quite well into PAR. This comprehensive framework takes the user through five major domains (*intervention characteristics, outer setting, inner setting, characteristics of individuals* and *process*) with several underlying constructs. Thus, it increases researcher and participant awareness of critical topics like the adaptability of an innovation or the setting’s implementation climate throughout an implementation project.

Several implementation strategies are available that aim to support and facilitate successful guideline use and to help overcome possible barriers during the implementation process. The *Cochrane Effective Practice and Organisation of Care* (*EPOC*) Review Group provided a framework that distinguishes four different types of interventions: professional, financial, organisational and regulatory interventions. Each type of intervention comprises a list of several implementation strategies [10]. These lists provide a valuable overview of possible strategies for meeting the different requirements of an implementation process and furthermore include strategies which vary from bottom-up (through participation) to top-down approaches (through instructions). The *EPOC* framework has been suggested for use in guideline implementation studies, either for planning the implementation process itself and/or for designing a study [11]. Yet, the framework does not specify the effectiveness of the individual implementation strategies.

**References**

1. Leykum LK, Pugh JA, Lanham HJ, Harmon J, McDaniel Jr RR. Implementation research design: integrating participatory action research into randomized controlled trials. Implement Sci. 2009;4:69. doi: 10.1186/1748-5908-4-69.
2. Greenhalgh T, Robert G, Bate P, Kyriakidou O, Macfarlane F, Peacock R. How to spread good ideas: a systematic review of the literature on diffusion, dissemination and sustainability of innovations in health service delivery and organisation. Report for the National Coordinating Centre for NHS Service Delivery and Organisation R&D (NCCSDO). 2004. [http://www.netscc.ac.uk/hsdr/files/project/SDO_FR_08-1201-038_V01.pdf].
3. Waterman H, Tillen D, Dickson R, de Koning K. Action research: a systematic review and guidance for assessment. Health Technol Assess. 2001;5:23.
4. Carr W, Kemmis S. Becoming critical. Education, knowledge and action research. Reprint, Victoria: Deakin University Press; 1990.
5. Smith A. An action research inquiry exploring the transfer of pain knowledge from a continuing education course into practice. PhD thesis. University of Stirling, Department of Nursing and Midwifery; 2008. [https://dspace.stir.ac.uk/bitstream/1893/474/1/A%20Smith%20-%20An%20action%20research%20inquiry%20ecploring%20the%20transfer%20etc.pdf].
6. Dick B. A beginner’s guide to action research. 2000. [http://158.132.155.107/posh97/private/research/methods-action-research/guide.pdf].
7. Kemmis S. Exploring the relevance of critical theory for action research: emancipatory action research in the footsteps of Jürgen Habermas. In: Reason P, Bradbury H, editors. Handbook of action research. 4th ed. London: Sage Publications; 2006. p. 94–105.
8. Damschroder LJ, Aron DC, Keith RE, Kirsh SR, Alexander JA, Lowery JC. Fostering implementation of health services research findings into practice. A consolidated framework for advancing implementation science. Implement Sci. 2009;4:50. doi: 10.1186/1748-5908-4-50.
9. Ilott I, Gerrish K, Booth A, Field B. Testing the consolidated framework for implementation research on health care innovations from South Yorkshire. J Eval Clin Pract. 2013;19:915–24.
10. EPOC. Cochrane Effective Practice and Organisation of Care Review Group (EPOC) data collection checklist. 2002. [http://epoc.cochrane.org/sites/epoc.cochrane.org/files/uploads/datacollectionchecklist.pdf].
11. Mäkelä M, Thorsen T. A framework for guidelines implementation studies. In: Thorsen T, Mäkelä M, editors. Changing professional practice. Theory and practice of clinical guidelines implementation. Copenhagen: Danish Institute for Health Services Research and Development; 1999. p. 23–53.
