# Supplementary material for: Effectiveness of multifaceted and tailored strategies to implement a fall-prevention guideline into acute care nursing practice: a before-and-after, mixed-method study using a participatory action research approach
Source: BMC Nurs. 2015 Mar 31;14:18. doi: 10.1186/s12912-015-0064-z (PMC4394413; doi:10.1186/s12912-015-0064-z)
Supplement: Additional file 2: — Nursing personnel involvement and applied implementation strategies. Description of nursing personnel involvement during the implementation process and of the implementation strategies used. [file 12912_2015_64_MOESM2_ESM.doc]

**Additional file 2: Nursing personnel involvement and applied implementation strategies**

Breimaier HE, Halfens RJG, Lohrmann C: **Effectiveness of multifaceted and tailored strategies to implement a fall-prevention guideline into acute care nursing practice: a before-and-after, mixed-method study using a participatory action research approach**

*Nursing personnel involvement*

A steering group comprising nursing personnel representatives was recruited from both departments: five representatives from the ASD and eight from the OD with HEB serving as moderator and advisor in each group. During six (ASD)/ seven (OD) monthly meetings between January and June/July 2011, the implementation of the Falls CPG was planned, necessary measures initiated and their progress continually evaluated. The results of the baseline data collection informed the course of action for each steering group. All graduate and assistant nurses not members of a steering group were given the opportunity to present their ideas and/or critiques, either in person during a steering group meeting or indirectly through their representatives. The main purposes of the steering group meetings were to determine:

- the problem of falls from the perspective of nursing personnel
- gaps in preventive measures already carried out by nursing personnel
- nursing personnel’s aims for guideline implementation and appropriate criteria for their measurement*
- successful implementation criteria from the participants’ point of view (for example, availability of necessary means/equipment for fall prevention)

*Nursing personnel’s aims included a reduction of falls and their effects; more knowledge regarding fall prevention, Falls CPG recommendations and on recording a patient fall; optimising patient care in fall prevention; consistent procedures in fall-preventive actions and documentation; having adequate means and staff to adhere to fall-prevention recommendations; environmental modification (barrier-free access to the shower for disabled and less mobile patients, doorframe ramp to the balcony); patient information for hospital stay including adequate slippers and personal walking aids; making fall-prevention efforts visible.

Furthermore, steering group meetings served to consecutively select and tailor adequate implementation strategies and to initiate appropriate measures, including:

- defining the content of an educational programme for each department
- compiling a reference book with unit-specific information including written materials specified to each unit; for instance core information to be given to patients regarding fall prevention or a list of commonly present drugs known to raise the risk of falls
- determining additionally necessary means/equipment to prevent falls and necessary environmental modifications
- determining the mode of audit and feedback with consideration of existing structures in both departments

All steering group members encouraged their team to use the evidence-based guideline recommendations in daily practice. Furthermore, they served as facilitators for their team by answering questions, and by collecting information, additional ideas and/or critiques that were consequently integrated into the ongoing process.

*Applied implementation strategies*

Six implementation strategies were tailored to the needs of each department, then applied and classified according to *EPOC* [1]:

1. Educational meetings: 3 identically-organised 90-minute lectures were held for OD nursing personnel. ASD nursing personnel attended short lectures (2 x 15 minutes) during two team meetings. Those who could not attend one of these meetings were informed individually by a steering group member.
2. Distribution of written materials: The reference books were made available to each unit and nursing personnel was obliged to act according to the compiled material as soon as the training was completed.
3. Local opinion leaders: Steering group members from both departments acted as motivators in their respective teams as soon as the steering groups were established.
4. Audit and feedback: Assessment of fall risks, nursing care planning and measures taken in daily practice were audited using the pre-existing structures of both departments. Within the ASD, audit and feedback were included into the weekly nursing ward rounds, and the nurse in charge of the respective patient received appropriate feedback from the ward manager. Within the OD, audit and feedback were included through regular audits of nursing records, and feedback was given to the team by the appointed nurse. After a fall, a report had to be written which was audited by the head nurse, who in turn gave feedback to the responsible nurse.
5. Adaptation of nursing record systems: Only minor adaptations were necessary in both departments and installed/executed prior to the educational meetings, for example by adding phrases like ‘patient information given’ to allow documentation measures to be performed quickly.
6. Changes in physical structure, facilities and equipment: Necessary equipment was purchased and environmental modification was initiated. These were used immediately upon receipt.

The first four implementation strategies were part of the *professional interventions* category as they were directed towards the nursing personnel. The latter two were part of the *organisational interventions* category [1]. Financial incentives could not be offered as there was no extra budget available for this project.

**References**

1. EPOC. Cochrane Effective Practice and Organisation of Care Review Group (EPOC) data collection checklist. 2002. [http://epoc.cochrane.org/sites/epoc.cochrane.org/files/uploads/datacollectionchecklist.pdf].
